# Deep Mutational Scanning of Viral Glycoproteins and Their Host Receptors

**DOI:** 10.3389/fmolb.2021.636660

**Published:** 2021-04-09

**Authors:** Krishna K. Narayanan, Erik Procko

**Affiliations:** Department of Biochemistry and Cancer Center at Illinois, University of Illinois, Urbana, IL, United States

**Keywords:** viral fusion protein, entry receptor, virus spike, deep mutational scan, selection, viral escape, mutational landscape

## Abstract

Deep mutational scanning or deep mutagenesis is a powerful tool for understanding the sequence diversity available to viruses for adaptation in a laboratory setting. It generally involves tracking an *in vitro* selection of protein sequence variants with deep sequencing to map mutational effects based on changes in sequence abundance. Coupled with any of a number of selection strategies, deep mutagenesis can explore the mutational diversity available to viral glycoproteins, which mediate critical roles in cell entry and are exposed to the humoral arm of the host immune response. Mutational landscapes of viral glycoproteins for host cell attachment and membrane fusion reveal extensive epistasis and potential escape mutations to neutralizing antibodies or other therapeutics, as well as aiding in the design of optimized immunogens for eliciting broadly protective immunity. While less explored, deep mutational scans of host receptors further assist in understanding virus-host protein interactions. Critical residues on the host receptors for engaging with viral spikes are readily identified and may help with structural modeling. Furthermore, mutations may be found for engineering soluble decoy receptors as neutralizing agents that specifically bind viral targets with tight affinity and limited potential for viral escape. By untangling the complexities of how sequence contributes to viral glycoprotein and host receptor interactions, deep mutational scanning is impacting ideas and strategies at multiple levels for combatting circulating and emergent virus strains.

## Introduction

The surfaces of enveloped viruses are decorated with glycoproteins that mediate attachment to host cells and fusion of the viral and cell membranes, allowing the viral genetic material to access the cytoplasm. By virtue of being exposed on the virus surface to the immune system and mediating the essential events of attachment and membrane fusion, viral glycoproteins are a primary target of neutralizing antibodies and inhibitors. Viral fusion and attachment glycoproteins possess structural similarities and common mechanisms within families but can have highly variable sequences that impact receptor usage, tissue- and host species-tropism, and antigenic properties (White et al., 2008; Banerjee and Mukhopadhyay, 2016; Murin et al., 2019). This sequence diversity has profound implications for antigenic change and spillover of new strains from animal reservoirs. Understanding the accessible sequence diversity (also referred to as the functional sequence space) of a viral glycoprotein, especially under selective pressures, helps in understanding and predicting the past and future of a virus’s natural evolution. In this review, we introduce and discuss deep mutational scanning as a generalizable methodology for learning about viral glycoproteins and their host receptors, in particular highlighting how the information impacts therapeutic and vaccine development.

## Deep Mutational Scanning as a Generalizable “Big Data” Technology

For decades, understanding the effects of mutations on a protein’s structure and function has involved targeted mutagenesis and the individual characterization of unique sequence variants. Much of what the field understands about protein sequence and its impacts on folding, stability, and function, originates from this classical approach. However, targeted mutagenesis is limited in scope by practical considerations, and is generally focused on a small number of candidate mutations that are hypothesized to be critical for activity, often based on similarities or differences with homologs. When critical residues in a protein sequence are unknown, an unbiased approach is needed. This led to the application of amino acid scanning mutagenesis, in which residues in the protein sequence are systematically and sequentially substituted to a specific amino acid. Most often this is by alanine substitutions (Cunningham and Wells, 1989), which illustrates the effect of removing an amino acid’s side chain, but other amino acids have also been explored as alternatives for scanning mutagenesis (i.e., using aspartic acid or glutamic acid for finding ligand binding interfaces) (Gray et al., 2017). Scanning mutagenesis has become a staple method for finding important functional or structural sites and has been extensively applied to viral glycoproteins and their receptors. This is well demonstrated by the example of human immunodeficiency virus 1 (HIV-1). The surface glycoprotein spike of HIV-1, Env, is a trimer that binds its primary receptor CD4 on a host cell, triggering conformational changes in Env that expose a binding site for a co-receptor, CCR5 or CXCR4. Co-receptor binding is followed by further conformational changes that mediate fusion of the HIV-1 envelope and host cell membranes (Merk and Subramaniam, 2013; Wang et al., 2020). This system has been extensively interrogated by alanine scanning, both in the viral spike (Lu et al., 2001; Jacobs et al., 2005; Jiang and Aiken, 2007; Walker et al., 2009, 2011; Sen et al., 2010; Li et al., 2011) and its receptors (Ashkenazi et al., 1990; Dragic et al., 1998; Rabut et al., 1998; Chabot et al., 1999), identifying key residues for expression, stability, physical interactions, conformational dynamics, cell entry, and interactions with monoclonal antibodies. Alanine substitution mutants have been quantitatively characterized in exceptional detail, such as determining changes in Env expression, proteolytic processing into its subunits, virus incorporation, receptor binding, and infectivity in culture. However, these scanning mutagenesis experiments do not fully account for the dependency on the chemical properties of an amino acid present at a certain position (Haddox et al., 2016; Dingens et al., 2017); findings may be very different depending on which of the 20 amino acids is chosen for a substitution. In contrast, deep mutational scanning may test all possible amino acid substitutions at each position, demonstrating how side chain properties and possible steric or electrostatic clashes influence the protein’s structure and function. This increase in scale is achieved by forgoing individual characterization of each mutation and instead using *in vitro* selections of variant libraries coupled with next generation sequencing to determine the effects of thousands of mutations in a single experiment.

In the past decade, deep mutational scanning has provided a more complete picture of a protein’s mutational landscape under controlled experimental conditions. In a typical deep mutagenesis experiment, a library is constructed that encompasses the mutations of interest, often by incorporating degenerate codons at each position in the cDNA sequence to encode all possible single amino acid substitutions. The variant library is then exposed to *in vitro* selection and changes in variant frequency are measured by deep sequencing. In this way, it becomes possible to characterize thousands of mutations simultaneously from a single selection experiment (Fowler et al., 2010; Fowler and Fields, 2014). Deleterious mutations are depleted while beneficial mutations are enriched, and the enrichment ratio for a given sequence variant acts as a proxy for relative phenotype. This qualitatively defines the phenotypic effects of each mutation at diversified positions in the variant library. If a series of parallel selections are performed with increasing stringency [for example, cells expressing libraries of protein variants are collected in parallel for increasingly higher expression levels (Matreyek et al., 2018) or for ligand binding at increasingly lower ligand concentrations (Adams et al., 2016)], then it is also possible to quantitatively determine phenotypic changes for a mutant protein based on trends going from low to high selection stringency. Deep mutagenesis can also be performed without using an *in vitro* selection, such as when protein activity is linked to transcription of a barcoded reporter (Jones et al., 2020), but these cases are not considered in this review where the focus is on selections of viral glycoproteins and their receptors.

The disadvantage of deep mutagenesis is that scale may be achieved at the expense of data accuracy, especially as the diversity of variants in the library becomes too large to be sufficiently sampled or the selections lack stringency to discriminate between variants of differing activities. This is again highlighted by an example from the study of HIV-1, where a deep mutational scan of Env based on virus infectivity in culture showed partial agreement with previous targeted mutagenesis of the receptor binding sites (Olshevsky et al., 1990; Basmaciogullari et al., 2002), but no correlation with prior alanine scanning in the Env gp41 subunit (Jacobs et al., 2005; Sen et al., 2010). There was also little correlation to natural sequence diversity in circulating strains (Haddox et al., 2016). These issues were due to low selection stringency that allowed an uncharacteristically high number of Env variants to persist during passaging, in addition to high noise in the data emphasized by poor agreement between independent experimental replicates (Haddox et al., 2016). However, despite these deficits, sequence features were still apparent, such as lower mutational tolerance in epitopes for broadly neutralizing antibodies (Haddox et al., 2016). Comparisons of earlier deep mutational scans of HIV-1 Env (Haddox et al., 2016; Heredia et al., 2019) with more recent investigations of SARS coronavirus 2 (SARS-CoV-2) spike and its receptor (Chan et al., 2020; Starr et al., 2020) have shown dramatic improvements in data quality, due to changes in selection strategies toward surface display technologies and more efficient sampling of smaller libraries. These more recent works withstood extensive validation by targeted mutagenesis of selected individual mutations. Furthermore, as discussed below, confidence in experimental mutational data is improved through the use of algorithms that bring other sources of information to bear on the problem, such as conservation among homologous sequences and consideration of chemicophysical properties of the amino acids being substituted (Weile et al., 2017; Shamsi et al., 2020). The relevance of deep mutagenesis to understanding viral glycoproteins and their receptors is therefore expected to grow as the technology matures.

Different selection strategies open possibilities for deep mutational scanning as a generalizable tool for studying different protein properties and activities, from solubility-activity relationships for protein engineering (Klesmith et al., 2017; Wrenbeck et al., 2017; Gupta and Varadarajan, 2018) to examining the molecular determinants of amyloid-β aggregation (Gray et al., 2019) to exploring pathogenic variants in the human genome (Stein et al., 2019). Moreover, deep sequencing has been used to analyze library selections of increasing sophistication and creativity. This has included selections for folded structure based on protease sensitivity (Rocklin et al., 2017), co-trafficking of subunits in oligomeric complexes (Park et al., 2019), protein-protein interactions both inside and outside the cell (McLaughlin et al., 2012; Procko et al., 2014; McShan et al., 2019), signaling (Jones et al., 2020), and processing of endogenous substrates (McShan et al., 2020), among other examples. In order to better combat current and new strains of circulating viruses, deep mutagenesis studies seek to mimic aspects of virus evolution in a laboratory setting, providing insights into the accessible sequence diversity for genetic drift, immune escape, and drug resistance. Deep mutational scanning reveals amino acid preferences of viral glycoproteins and how those preferences shift in the context of applied selective pressures, such as antibodies or drugs, and can further inform structural understanding and rational design of therapeutic or prophylactic interventions, for example, by revealing vulnerable epitopes.

## Selection Strategies for Understanding Sequence Diversity and Evolution of Viral Glycoproteins

**FIGURE 1:**
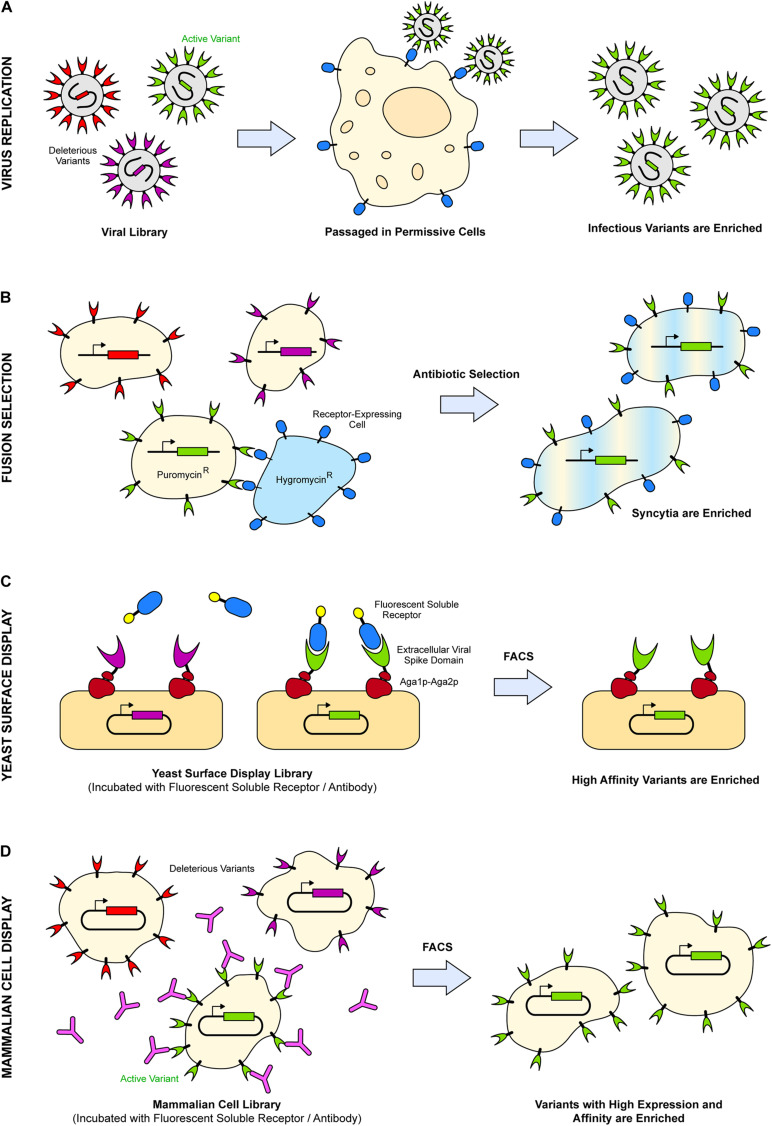
Examples of selection strategies used in deep mutational scans of viral spike proteins. **(A)** Infectious virus variants (green) are enriched after passaging through a permissive cell line expressing host target receptors (blue). **(B)** Syncytia are enriched upon fusion of cells expressing variants of viral fusion protein (pale yellow cells) and cells expressing the entry receptor (pale blue cells) in the presence of two different antibiotics. **(C)** Soluble extracellular domains of the viral spike are displayed on the yeast cell wall via Aga1p–Aga2p (dark red). After fluorescence activated cell sorting (FACS), these viral proteins (green) are enriched if they possess high binding affinity and/or expression to fluorescent partners, such as soluble receptors (blue). **(D)** After FACS, mammalian cells expressing viral spike proteins (green) are enriched if they are highly expressed and bind with high affinity to fluorescently labeled antibodies (pink) or soluble receptors.

The requirements for deep mutagenesis are (i) a library of diverse sequence variants, (ii) a suitable host that links phenotype to genotype, and (iii) a selection strategy (Fowler et al., 2010; Fowler and Fields, 2014). The simple fulfillment of these requirements by using viruses and infected cells as the hosts and virus replication in culture as the selection (Figure 1) have propelled deep mutagenesis to the forefront of virology within the past decade. The general approach of tracking replication of virus variants through deep sequencing has been used to extensively characterize the glycoproteins of two viruses highly relevant to public health: HIV-1 (Haddox et al., 2016, 2018; Dingens et al., 2017, 2019a,b) and influenza A (Thyagarajan and Bloom, 2014; Wu et al., 2014, 2017a, 2020; Doud and Bloom, 2016; Canale et al., 2018; Lee et al., 2018). There have also been many efforts within the past few years to apply this selection strategy and others more broadly to other viruses, including murine leukemia virus (MLV) (Salamango et al., 2016), Zika virus (ZIKV) (Gong et al., 2018; Setoh et al., 2019; Sourisseau et al., 2019), and most recently, SARS-CoV-2 (Greaney et al., 2020; Linsky et al., 2020; Starr et al., 2020; Chan et al., 2021). Below, we introduce and discuss examples of selection strategies used for deep mutagenesis of viral glycoproteins.

To determine how mutations impact replicative fitness, viral libraries are first prepared by transfecting a virus production line with plasmids encoding variants of the spike protein and transducing with any helper virus as necessary. The produced virus particles in the supernatant are then passaged through a permissive cell line expressing the relevant host receptors (Figure 1). Infectious virus variants are enriched, while deleterious ones are depleted. This is the selection strategy used in the aforementioned study of HIV-1 Env, finding residues crucial for expression, folding, and receptor binding despite data noise and low selection stringency (Haddox et al., 2016). Similarly, the observed phenotypes of influenza A hemagglutinin (HA) variants using the same selection strategy of virus replication in culture were consistent with reported phenotypes of previously characterized mutants (Wu et al., 2014). For influenza A, spikes composed of three HA subunits on the viral envelope bind sialylated glycans on host cells to mediate endocytosis, followed by acid-induced conformational changes of the HA trimer within the endosome that drive fusion of viral and cell membranes (Dou et al., 2018; Russell et al., 2018). Whether through single-nucleotide mutations (Wu et al., 2014) or through the full breadth of mutations available at each codon (Thyagarajan and Bloom, 2014), the studies elucidated that influenza HA possesses a high inherent mutational tolerance which, in conjunction with external selective pressures, drives the rapid evolution of the virus. The selection strategy illuminates the constraints on a glycoprotein’s inherent evolutionary capacity, at least in the context of replication in cell culture, creating specific sequence-fitness links at each site. However, virus replication requires the surface glycoproteins to be properly folded, incorporate into virus particles, bind host entry receptors, and undergo complex dynamic changes that drive fusion of viral and host cell membranes, and it is difficult to know which process is disrupted by a deleterious mutation.

Multiple selection strategies can be implemented for the same deep mutagenesis library to determine the effects of mutations separately on different properties of a viral fusion protein or glycoprotein. Salamango et al. (2016) utilized three different selection strategies on deep mutational scans of MLV Env for infectivity, Env fusion activity, and incorporation into viral particles. The mutational landscapes for the strategies were compared to filter out how specific sites and their mutations influence each selected property. Since the fusion selection strategy was decoupled from the formation and passaging of virus particles, mutations enriched for Env fusion may arise that do not correlate with infectious, well-formed particles. For the fusion selection, a library of mammalian cells expressing Env variants were incubated with a cell line expressing the entry receptor. The two cell populations had resistance to two different antibiotics, such that the fused syncytia were selected in the presence of both antibiotics (Figure 1). Mutations in MLV Env were discovered with defective infectivity because of poor incorporation, despite the Env mutants remaining active for membrane fusion. These MLV Env mutants were poorly incorporated into pseudotyped HIV-1 particles, possibly due to changes in lipid interactions at assembly sites (Salamango et al., 2016). Pseudotyping allows for the manipulation of cell type tropism through the incorporation of envelope glycoproteins from one virus into alternative virus backgrounds. It allows for the ability to study fusion proteins from highly virulent strains in backgrounds of reduced safety risk. In addition to a diverse range of clinical and non-clinical applications, pseudotyped viruses can be used to validate predictions from deep mutagenesis studies, as was done with the mutational landscape of SARS-CoV-2 spike glycoprotein S (Greaney et al., 2020; Starr et al., 2020).

Another selection strategy decoupled from virus replication is yeast surface display. It involves the expression of a soluble protein fragment, such as an extracellular domain of a viral spike, fused with yeast adhesion factor, Aga2p, which complexes with Aga1p for display on the yeast cell wall (Gai and Wittrup, 2007). Yeast transformed with libraries encoding protein variants are fluorescently labeled for detection of expression or activity and sorted by fluorescence-activated cell sorting (FACS) (Figure 1). For viral protein variants, surface expression levels can be detected using epitope tags, while binding activity is assessed with fluorescently labeled antibodies or soluble receptors. Yeast display has a fast turnaround for generating data, and unless there is a pressing need to understand the effects of viral glycoprotein mutations on infectivity, it presents a powerful approach for the rapid development and testing of therapeutics (Gaiotto and Hufton, 2016; Linsky et al., 2020; Starr et al., 2020). The benefits of yeast surface display for quickly assessing mutational tolerance were demonstrated in the months following the emergence and global spread of SARS-CoV-2. SARS-CoV-2 spike protein S forms a trimeric fusion protein that engages angiotensin converting enzyme 2 (ACE2) on host cells as an entry receptor (Hoffmann et al., 2020; Lan et al., 2020; Wrapp et al., 2020; Yan et al., 2020; Zhou et al., 2020). The binding site is located on a receptor-binding domain (RBD) of S, which was shown through a deep mutational scan of the RBD expressed on the yeast surface to have high mutational tolerance with respect to both expression and ACE2 binding affinity (Starr et al., 2020). The findings were confirmed by a later deep mutational scan of the RBD in the context of full-length trimeric S expressed on human cells (Chan et al., 2021) and align with comparative bioinformatics of S proteins from SARS-associated betacoronaviruses isolated from bats, where diversity within the ACE2-binding site of the RBD is surprisingly high (Frank et al., 2020). This diversity is possibly due to an “arms race” between S and ACE2 co-evolution; ACE2 is highly polymorphic across different bat species (Guo et al., 2020).

Mutations at several sites in the SARS-CoV-2 RBD, but especially N501, were predicted by deep mutational scanning and confirmed by targeted mutagenesis to enhance binding to the ACE2 receptor (Starr et al., 2020; Chan et al., 2021), potentially creating opportunities for the virus to become more infectious or partially resistant to therapeutics blocking the RBD-ACE2 interaction. The N501 site has since gained notoriety for emerging in at least three separate variant lineages with increased transmissibility, B.1.351 in South Africa (Tegally et al., 2020), P.1 in Brazil (Faria et al., 2021), and B.1.1.7 in England (Leung et al., 2021; Volz et al., 2021). Even though much is still unconfirmed about the characteristics of these variants, their emergence lends support to the predictive power of deep mutational scanning. The deep mutagenesis data, in combination with the observed natural diversity among SARS-related coronaviruses, raise concerns that the spike sequence has the capacity to drift substantially, potentially causing changes in dominant antigenic epitopes and escape from immunity (Wibmer et al., 2021). SARS-CoV-2 has a moderate mutation rate estimated at 10^–3^ substitutions per site per year (Candido et al., 2020), but has shown rapid accumulation of mutations in farmed mink (Oude Munnink et al., 2021) and an immunocompromised patient (Choi et al., 2020).

Major limitations of yeast display are (i) that only soluble extracellular domains are expressed that lack transmembrane regions, (ii) that some complex proteins do not properly fold on the yeast surface, and (iii) that yeast lack terminally sialylated *N*-glycans found on human membrane proteins (Wildt and Gerngross, 2005), which can impact interactions with glycan-dependent antibodies (Cohen et al., 2015). Although yeast display is advantageous for rapid characterization of viral glycoproteins, it notably excludes considerations required for expression and folding in human cells. A selection strategy that accounts for these considerations is mammalian cell surface display. Genes encoding spike variants are expressed in mammalian cells using transfection or transduction conditions that yield one protein variant per cell (Forsyth et al., 2013; Steichen et al., 2016; Bruun et al., 2017; Kulp et al., 2017; Matreyek et al., 2017, 2020; Heredia et al., 2018; Procko, 2020). This links the cell phenotype to a single variant genotype. Cells are incubated with fluorescently labeled antibodies or soluble receptors and sorted by FACS, so cells expressing spike variants with high expression and high binding affinity to the fluorescent partner are enriched (Figure 1). In addition to deep mutational scanning of the isolated SARS-CoV-2 RBD by yeast display, it has been scanned in the context of full-length S expressed at the plasma membrane in human cells (Chan et al., 2021). The effects of mutations were qualitatively similar to results from yeast surface display, and the two methods therefore reinforce the major conclusion that the viral spike is able to tolerate high mutational diversity while maintaining expression and ACE2 binding activity (Starr et al., 2020; Chan et al., 2021). Conflicts between the data sets were mostly confined to a small number of RBD residues that are buried in the major prefusion conformation of S yet are exposed when the isolated RBD is expressed on yeast, as well as higher mutational tolerance for receptor binding in the human cell data set, possibly due to differences in avid binding of dimeric ACE2 receptors between the two systems. Both studies ignore mutations in the viral spike outside the RBD that may influence escape from antibodies or modulate receptor binding through allostery or epistasis, for example, by increasing dynamic exposure of the RBD for receptor recognition as occurs in the D614G virus variant (Ozono et al., 2021; Xu et al., 2021). Future work should be dedicated toward understanding the mutational landscape of the entire S glycoprotein for folding/expression, ACE2 binding, infectivity, and interactions with monoclonal antibodies targeting domains other than the RBD. Indeed, previous work with HIV-1 Env has shown how mutations distal from the binding interface impact receptor recognition through conformational effects (Heredia et al., 2019).

## Predicting Virus Escape From Antibodies and Drugs Through Deep Mutational Scanning

External selective pressures influence variant fitness and spike protein adaptation and are, thus, required for recapitulating aspects of natural evolution in deep mutagenesis studies. Furthermore, viruses can adapt to prevailing selective pressures from therapeutics or the immune system, posing a challenge to therapeutic and vaccine development. By performing selections *in vitro* on comprehensive variant libraries, one can search for escape mutations accessible to the virus. Expression systems of viral proteins, whether it be viruses (Doud and Bloom, 2016; Dingens et al., 2017, 2019a,b; Wu et al., 2017a; Phillips et al., 2018; Sourisseau et al., 2019), yeast (Gaiotto and Hufton, 2016; Greaney et al., 2020; Linsky et al., 2020), or mammalian cell surface display (Chan et al., 2021), are incubated with therapeutics before passaging through cell culture for the former and before FACS sorting for the latter two (Figure 1). However, escape mutations predicted by surface display strategies may not account for a loss of replicative fitness, unlike selections with live virus, although they permit a more comprehensive assessment of mutational effects without the constraint of viral replication. For example, while the influenza A H1 HA stem is moderately conserved in studies with live virus, yeast displayed HA identified mutations at and around the fusion peptide in the HA stem that preserved expression but led to a reduction in nanobody binding. The fusion peptide is an essential motif for mediating virus-cell membrane fusion, so mutations in the vicinity of this site, while reducing nanobody binding, also reduce infectivity (Gaiotto and Hufton, 2016). An approach to address the pleiotropic effects of mutations has been to test surface displayed libraries of viral spikes for binding to host receptors versus antibodies (or antibody-like molecules), thereby isolating putative escape variants that selectively lose antibody affinity while maintaining tight receptor recognition (Greaney et al., 2020; Linsky et al., 2020; Chan et al., 2021; Starr et al., 2021). This strategy has isolated mutations in S of SARS-CoV-2 that mediate escape from monoclonal antibodies used clinically for the treatment of COVID-19 (Starr et al., 2021). Epitopes that are well-established as sites for antibody neutralization may have a high capacity to tolerate mutations. Therefore, mapping escape mutations to atomic-resolution structures defines important sites for the improvement of a therapeutic’s design and identifies virus variants that might yet emerge in nature if the therapeutic is widely used.

Deep mutational scans in the presence of different external pressures can reveal conserved sites for the rational design of therapeutics, such as universal antibodies (Doud and Bloom, 2016; Gaiotto and Hufton, 2016; Phillips et al., 2018), and can support creative treatment strategies to restrict a virus’s capability for escape. For example, by comparing potential escape mutations specific to different antibodies, mixtures of antibodies can be chosen that bind non-competing epitopes and do not share common escape mutations. These antibody cocktails exploit the orthogonality of escape mutations to suppress the emergence of resistance (Gaiotto and Hufton, 2016; Dingens et al., 2019b; Greaney et al., 2020). While escape mutations to antibodies are often found at the epitope, deep mutational scanning also identifies escape mutations at sites that do not interact directly with an antibody but rather influence binding through allostery and conformational shifts. A deep mutational scan focused on two loops of HIV-1 Env that interact with CD4 found mutations that increase virus fitness in culture by changing Env conformational dynamics (Duenas-Decamp et al., 2016). Trimeric Env exists in an equilibrium of conformational isomers and undergoes a complex dynamic process of structural changes during fusion. Mutations that alter these dynamics provide a mechanism for escape from conformation-dependent antibodies (Dingens et al., 2019b). Mutations to residues that are separated by a small number of structural contacts to the epitope, or mutations that alter glycosylation patterns, also provide opportunities for escape from bnAbs (Dingens et al., 2019b). In contrast to antibodies, engineered decoy receptors resemble the host receptors, aside from mutations to improve binding or specificity to the viral glycoproteins of interest. The viral spikes may not be able to develop escape mutations without a simultaneous loss of affinity to their membrane-bound target receptors. This has already been demonstrated in human cells expressing a library of RBD-focused variants in full-length SARS-CoV-2 S, sorted in the presence of competing wildtype and engineered ACE2 decoy receptors. Escape mutations in S discriminating against an engineered, high affinity, soluble decoy receptor weren’t found, and if used as a therapeutic, the virus is unlikely to become resistant (Chan et al., 2021). However, viruses are not limited to single substitutions for escape mutations, and epistatic relationships can be complex when multiple sites are mutated together. These kinds of studies are therefore best considered as supplementing, rather than replacing, classical selections for escape variants, in which viruses are passaged in the presence of antiviral agents (e.g., antibodies, drugs) to promote the emergence of resistance on an accelerated time scale *in vitro* (Fellinger et al., 2019; Baum et al., 2020; Higuchi et al., 2020).

## Considerations of Epistasis, Phylogeny, and Cell Type in Deep Mutational Scanning

Single substitutions in deep mutational scanning of viral proteins cannot capture all the possibilities for immune escape and natural evolution. Early deep mutagenesis studies of influenza HA primarily focused on single amino acid substitutions and did not account for epistasis when multiple sites are mutated in combination, which can lead to shifts in amino acid preferences and alter the tolerance of sites (Hilton and Bloom, 2018). These scans reveal many single substitutions that cause deleterious effects on expression or receptor binding, but functionality and fitness might recover when substitutions are combined. For example, mutations in a loop at the receptor binding site of HA show extensive epistasis, including cases where two mutations that are deleterious on their own are neutral when combined together (Wu et al., 2017a). These epistatic effects substantially increase the functional sequence space compared to what additive effects of single mutations would predict. Epistasis between residues demonstrates a fundamental limit to focusing on single substitutions in deep mutational scanning to accurately predict how viruses might respond to new pressures, but this focus is often experimentally necessary to reduce library diversity for sufficient sampling of variants during selection. In the study by Wu et al. (2017a), library diversity was instead constrained by only allowing combinations of mutations at a small subset of residues.

As amino acid substitutions accumulate in viral glycoproteins in response to selective pressures, epistatic interactions can modulate the local mutational landscape and may shift amino acid preferences, stabilizing previously deleterious mutations (Wu et al., 2020). This may lead to the entrenchment of these substitutions and may even cause wildtype reversion to become unfavorable (Haddox et al., 2018). If strains are separated over a period of time, the accumulation of sequence differences can lead to substantial divergence between their mutational landscapes (Wu et al., 2020). Deep mutational scans of Env proteins from different HIV-1 strains demonstrate, despite substantial overlap, that amino acid preferences at some sites are strain-specific and therefore highly dependent on the background sequence (Haddox et al., 2018; Heredia et al., 2019). Strain-specific differences shape both antigenicity and mutational tolerance, so escape mutations from antibodies may be strain-specific as well. In the case of rapidly mutating viruses, like influenza A, understanding mutational tolerance of the glycoprotein may be context dependent because of evolutionary differences between subtypes. Within the phylogenetic tree of influenza HA, all subtypes share a highly conserved structure and perform the same function of binding to sialic acids on host cells. However, amino acid sequence identity between subtypes can be as low as 38% (Lee et al., 2018) and the subtypes have distinct amino acid preferences (Canale et al., 2018; Hilton and Bloom, 2018). For instance, in a deep mutational scan of HA subtype H1, the head domain is more mutationally tolerant than the stalk/stem domain, but a deep mutational scan of HA subtype H3 reveals the reverse (Lee et al., 2018). The implication is that studying the molecular evolution of one subtype is less useful for understanding the evolution and predicting escape mutations in others, an important caveat that may extend into comparing variants within the same subtype.

By carefully choosing the cell line that acts as the host for virus replication, deep mutagenesis can identify mutations in viral proteins that influence host interactions and adaptation (Ashenberg et al., 2017; Setoh et al., 2019; Shirleen Soh et al., 2019; Sourisseau et al., 2019). Zika virus (ZIKV) has received significant interest in recent times due to its broad tissue tropism that permits placental transmigration and neurological defects in a developing fetus (Castanha and Marques, 2020). Compared to HIV-1 Env and influenza HA, much less is known about the natural evolution of ZIKV envelope (E) protein, and host entry receptors and attachment factors remain unclear (Sirohi and Kuhn, 2017). A deep mutational scan of ZIKV E protein has indicated that its surface-exposed regions, except for the fusion loop and hypervariable glycan loop, display higher mutational tolerance in the experimental scan compared to alignments of natural sequence variants. ZIKV E protein is likely less tolerant of mutations in nature where it faces stronger selective pressures during replication in hosts that actively mount immune responses, in addition to undergoing part of its replication cycle in *Aedes* species mosquitoes (Sourisseau et al., 2019). Furthermore, most natural ZIKV variants were sequenced from human patients in just the last few years and are therefore closely related on a short evolutionary timescale; the available functional sequence space will therefore be under-sampled in natural isolates. Other deep mutational scans have identified E variants that specifically enhance ZIKV replication in either mosquito or primate cell lines, possibly due to the effects of mutations on temperature-sensitive structural transitions (replication in insect cells occurs at a lower temperature) (Setoh et al., 2019) or the removal of glycans that specifically aid human cell infection via the lectin DC-SIGN (Gong et al., 2018). Performing deep mutagenesis studies of viruses with zoonotic origins in multiple cell types can inform our understanding of how adaptation and evolution leads to spillover from animal reservoirs.

## Deep Mutagenesis-Guided Immunogen Engineering

Deep mutational scanning of viral glycoproteins not only provides insight into possible evolutionary pathways the virus may take, especially in the presence of drugs and antibodies, but it may also guide the engineering of the glycoproteins as optimized immunogens to promote broad and effective immunity. Subunit vaccines are composed of purified viral glycoproteins or their components, which stimulate the clonal expansion, affinity maturation, and immunoglobulin class switching of naive B cells expressing low affinity receptors. It is often critical that the immunogenic glycoproteins are conformationally stable and pure, and if necessary, bind rare germline B cell receptors that are able to mature into the most potent and broad neutralizing antibodies (Medina-Ramírez et al., 2017; Stamatatos et al., 2017; Ringel et al., 2018). These concepts have been particularly well demonstrated in the deep mutational scanning and engineering of immunogens for HIV-1, where vaccines have thus far failed to elicit a broadly protective humoral response against diverse virus strains.

Human immunodeficiency virus 1 Env adopts a range of conformational states, with most broadly neutralizing antibodies (bnAbs) targeting a so-called “closed” conformation in which strain-specific epitopes are hidden (Medina-Ramírez et al., 2017; Wang et al., 2020). Researchers have therefore placed priority on engineering soluble Env proteins that fold as stable closed trimers for optimum presentation of epitopes that may elicit broad protection. By having selections based on high affinity binding to conformation-dependent bnAbs, deep mutational scans of Env expressed on human cells have been able to identify mutations that stabilize the closed trimeric state and optimally present bnAb epitopes (Steichen et al., 2016; Kulp et al., 2017; Heredia et al., 2019).

Broadly neutralizing antibodies targeting HIV-1 Env are extensively mutated from their germline antibody genes, which have low affinity for Env and are therefore poorly primed by standard Env immunogens (Stamatatos et al., 2017). To overcome this barrier toward eliciting bnAbs in a naive individual, deep mutagenesis of Env trimers expressed on human cells (Steichen et al., 2016; Kulp et al., 2017) or of a highly engineered Env fragment displayed on yeast (Jardine et al., 2016) have aided the engineering of immunogens that tightly bind and prime naive B cells expressing relevant germline antibody precursors. One of these engineered immunogens is being evaluated in a phase I clinical trial (ClinicalTrials.gov Identifier: NCT03547245). Indeed, it is possible to engineer a series of immunogens that coax antibody maturation down a lineage toward potent and broad HIV-1 neutralization (Escolano et al., 2016; Steichen et al., 2016). The first immunogens in the vaccine regimen have the highest number of mutations from native Env and bind tightly to the necessary germline B cell receptors. Subsequent immunogens have successively fewer mutations and more closely resemble native Env. At each step, B cells are activated that bind engineered immunogens that ultimately match Env on native virus particles.

## Deep Mutagenesis of Host Receptors: Implications of Human Polymorphisms and Engineered Receptors as Therapeutics

In examinations of viral fitness, one cannot exclude the essential roles that host proteins play and how host genetic diversity may also impact infection and disease. Less attention has been given to deep mutational scanning of host proteins due to experimental challenges, since the underlying selection strategies can no longer be based on passaging of viral libraries. Instead, sequence diversity must be encoded in libraries of the host proteins that are expressed in mammalian cells or at the surface in yeast or mammalian cell display platforms. Host proteins with dedicated roles fighting viruses, such as antiviral restriction factors, have undergone co-evolution with viruses, a challenging feat due to vast differences in host versus virus evolutionary timescales. A deep mutational scan of TRIM5α, an antiviral restriction factor, has shown that its viral capsid-binding loop exhibits a very high mutational tolerance, which supports the main purpose of this unstructured loop to rapidly evolve against emergent retroviruses and hinder their propagation (Tenthorey et al., 2020). By comparison, host entry receptors generally have important physiological functions and are therefore often highly conserved. Instead of understanding their natural evolution, deep mutagenesis of virus entry or attachment receptors serves other important research objectives. How does receptor sequence diversity influence species tropism? Do some receptor polymorphisms make the host more or less susceptible to infection? Can mutational landscapes of receptors guide structural modeling and therapeutic engineering? These are questions that have only begun to be explored and offer rich areas for future investigation.

Two deep mutational scans of human ACE2, one from full-length protein expressed in human cells (Chan et al., 2020) and the second from surface display of the isolated ACE2 protease domain on yeast (Heinzelman and Romero, 2020), provide a near-comprehensive overview of how single substitutions contribute to SARS-CoV-2 S protein affinity. The deep mutational scans confirmed the structurally characterized S binding interface (Lan et al., 2020; Yan et al., 2020) and identified distal sites that affect binding, implicating conformational dynamics associated with ACE2 enzymatic activity in S affinity (Heinzelman and Romero, 2020). The mutational landscapes may contribute to our understanding of how ACE2 polymorphisms affect susceptibility to COVID-19 and for identifying at risk groups. ACE2 has a number of allelic variations within or near to the viral spike binding site. Because it is an X-linked gene, ACE2 polymorphisms may be of particular consequence in males (Gemmati et al., 2020). However, any predictions will need to be validated clinically by genetic studies.

**FIGURE 2:**
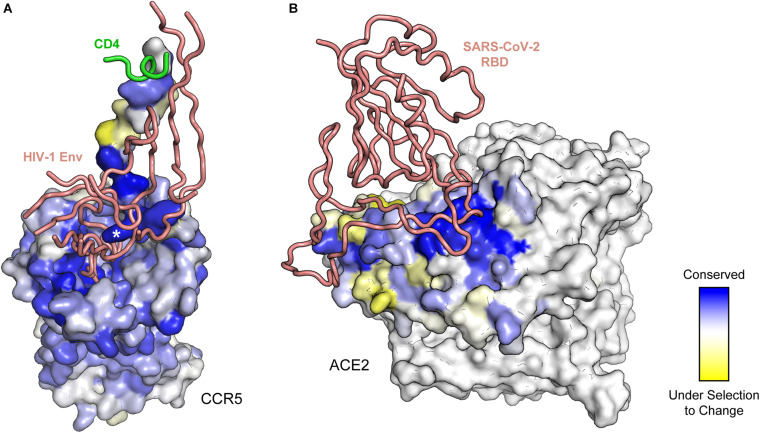
Deep mutagenesis of entry receptors identifies critical binding residues for viral fusion proteins. **(A)** Conservation from a deep mutational scan of the human immunodeficiency virus 1 (HIV-1) co-receptor CCR5 for interacting with CD4-bound Env is mapped to the structure (PDB 6MEO). For clarity, only residues of Env (peach ribbon) and CD4 (green ribbon) within proximity of CCR5 are shown. Critical CCR5 residues are blue, while residues that are under selection to change are yellow. The asterisk denotes CCR5 sulfotyrosine-14. **(B)** Deep mutagenesis data of human ACE2 (colored from blue for conserved to yellow for residues under selection to change) binding to the RBD of SARS-CoV-2 (peach ribbon) is mapped to structure (PDB 6M17). In the selection, residue conservation at the interface is bipartite, with one subsite on ACE2 (in dark blue) having very low mutational tolerance.

Deep mutational scanning can be used to map protein-protein interfaces (Figure 2) and guide structural modeling. Several groups have developed methods to glean structural information from deep mutagenesis and have shown how the data can be used to model both monomeric and oligomeric proteins (Park et al., 2019; Rollins et al., 2019; Schmiedel and Lehner, 2019; Fantini et al., 2020). These methods may be particularly useful for modeling complexes between viral glycoproteins and their host receptors when individual structures are known but the assembled complex is not, although we are unaware of any such studies at this time. Deep mutational scanning of CCR5, a co-receptor for HIV-1 Env, mapped critical receptor residues for Env binding (Heredia et al., 2018), but the data were of insufficient quality to enable accurate modeling. However, a retrospective comparison of the deep mutagenesis data to the cryo-EM structure, which was later solved (Shaik et al., 2019), demonstrates excellent agreement (Figure 2A). In particular, an exposed sulfonated tyrosine (sTyr-14) side chain from the receptor becomes buried in a deep pocket of Env; sTyr-14 is one of the most highly conserved residues for binding in the mutational scan. In another example, the structure for a trimeric glycoprotein complex from human cytomegalovirus (HCMV) binding its receptor, PDGFRα, was unknown beyond exceedingly low resolution cryo-EM images (Kabanova et al., 2016). Deep mutational scanning illustrates that HCMV trimer binding is largely resistant to single amino acid substitutions within two domains of the PDGFRα receptor (Park et al., 2020). Consequently, the PDGFRα mutational landscape failed to unambiguously highlight a surface epitope for modeling. A recently solved high-resolution structure of PDGFR-bound HCMV trimer shows why; the binding interface is extensive and spread over three receptor domains, such that disruption of interactions by any one receptor domain has minimal impact on HCMV trimer binding (Kschonsak et al., 2021).

An application of biochemical insights from deep mutagenesis is the optimization and engineering of soluble receptors as potent antivirals. Soluble decoy receptors present a unique opportunity for neutralizing viruses with little opportunity for the emergence of escape mutations. However, soluble decoy receptors can have problems with specificity and affinity that limit their efficacy *in vivo*. To again use the example of the HCMV glycoprotein trimer and its receptor PDGFRα, soluble PDGFRα ectodomain potently blocks virus entry at low nanomolar concentrations, yet the receptor also competitively binds four endogenous host factors involved in growth factor signaling, limiting its safety and efficacy as a therapeutic (Kabanova et al., 2016; Stegmann et al., 2017; Wu et al., 2017b). Using deep mutational scanning based on a competition selection between HCMV trimer and endogenous PDGFRα ligands, receptor mutations were screened that maintain tight virus binding and potent neutralization, while eliminating unwanted off-target interactions. These have been termed “orthogonal” receptors, as they are virus-specific and no longer participate in (i.e., are orthogonal to) the receptor’s normal biology (Park et al., 2020). This solves the specificity problem in the absence of an atomic resolution structure that could otherwise guide rational protein engineering.

In contrast to the specificity problem of soluble PDGFRα, the endogenous activity of soluble ACE2 acts as a negative regulator of the renin-angiotensin system to protect against lung injury in animal models of inflammation and infection (Imai et al., 2005; Treml et al., 2010; Zou et al., 2014). Soluble ACE2 catalyzes the proteolytic turnover of vasoconstrictive peptide hormones, which may offer direct relief from COVID-19 symptoms, and it is under evaluation in a phase II clinical trial (ClinicalTrials.gov Identifier: NCT04335136). However, wildtype soluble ACE2 binds S of SARS-CoV-2 with only moderate nanomolar affinity (Shang et al., 2020), significantly lower than affinity matured antibodies. Optimization of soluble ACE2 as a therapeutic is therefore not a problem of specificity but of affinity. Deep mutational scanning identified multiple mutations in ACE2 that enhance S binding, which in combination allow soluble ACE2 to achieve picomolar affinity (Chan et al., 2020). The neutralization potency of the engineered decoy receptor rivals monoclonal antibodies (Chan et al., 2020) and broadly binds with tight affinity to the RBDs of human SARS-CoV-1 and -2 as well as related bat coronaviruses (Chan et al., 2021). By resembling the natural receptor, the soluble decoy therefore achieves breadth for SARS-associated viruses.

In a similar vein, deep mutagenesis of computationally-designed proteins has been used to optimize their affinities and specificities for broad and potent antiviral activity. Small, hyperstable proteins can be designed that mimic natural entry receptors or monoclonal antibodies, but due to the immense freedom to explore structural and sequence space, the designed proteins may barely resemble the natural complexes from which they are inspired. In theory, proteins can be designed to bind any exposed surface on the viral target protein, and design can be focused to vulnerable epitopes that are conserved or essential for virus infection (Fleishman et al., 2011). Deep mutational scanning improves upon the computationally-designed proteins, which generally have only moderate affinity and require optimization (Whitehead et al., 2012). With this approach, one can optimize inhibitory “designer” proteins for high affinity and specificity to viral glycoproteins as well as orthogonality to host factors (Whitehead et al., 2012; Procko et al., 2014; Cao et al., 2020; Linsky et al., 2020). Moreover, in contrast to antibodies and soluble receptors, the designed proteins do not require expression in mammalian cells for proper folding and glycosylation, but can be generated in large quantities from bacteria (Cao et al., 2020; Linsky et al., 2020). Deep mutagenesis has been used to improve designed inhibitors targeting the proteins of influenza A (Whitehead et al., 2012), Epstein-Barr virus (Procko et al., 2014), and SARS-CoV-2 (Cao et al., 2020; Linsky et al., 2020).

## Limitations of Deep Mutagenesis and Avenues for Future Advancement

Deep mutagenesis data sets have inherent noise from multiple sources: insufficient experimental sampling of variants in the library, errors with accurately replicating collection gates associated with FACS-based selections, low signal-to-noise, and/or selections that are not properly stringent to discriminate between mutants of differing activities. Furthermore, epistatic interactions are often only assessed between a small number of sites to keep library diversity manageable (Wu et al., 2017a, 2020; Zhang et al., 2020) or are missed entirely in mutational scans based on single amino acid substitutions. As an alternative, statistical and computational methods are increasingly capable of accurately predicting mutational effects. Most popular are probabilistic models for describing sequence variation and fitness based on the Potts model from statistical physics, which incorporate both site-specific constraints on amino acid identity plus all of the possible pairwise constraints that describe covariation or coupling between pairs of positions (i.e., the degree to which amino acid identities at two positions are co-dependent) (Hopf et al., 2017; Levy et al., 2017). This information is extracted from alignments of homologous sequences, with the assumption that pairwise constraints between two positions are predictive of higher order couplings up to the entire sequence. The model can be improved if additional structure in sequence families, due to higher order epistasis not captured by pairwise constraints, is considered (Riesselman et al., 2018). The methods have accurately (and impressively) captured aspects of viral protein evolution in the clinic, especially for HIV-1, including drug resistance and escape by HIV-1 proteins from cellular and humoral immunity (Ferguson et al., 2013; Mann et al., 2014; Barton et al., 2016; Flynn et al., 2017; Louie et al., 2018; Biswas et al., 2019; Zhang et al., 2020). The pairwise couplings are critical to model accuracy, and the likelihood of an escape mutation occurring is heavily influenced by epistatic interactions with the background sequence (Barton et al., 2016). For example, if the background sequence has residues that are negatively coupled to an escape mutation, then more time is required for escape to occur as compensatory mutations must also accumulate. While surface glycoproteins have been less studied, modeling of the fitness landscape of HIV-1 Env has shown that, when considering both single site diversity and pairwise couplings, the protein surface is sparsely populated by sites that incur a large fitness penalty when mutated (Louie et al., 2018). This means antigenic epitopes tend to contain few residues that are truly conserved hidden amongst variable positions, emphasizing the difficulties for an antibody to achieve breadth.

The power of these statistical models derives from the enormous wealth of sequence information available from which to infer site-specific constraints and couplings. Where they triumph over deep mutational scanning is in their consideration of epistatic networks, yet they are also biased by sequence variation that occurs during natural evolutionary processes and may not provide mechanistic insights. However, models for predicting the effects of mutations, including but not limited to Potts probabilistic models, can be benchmarked or trained with experimental data, leading to more accurate predictions (Weile et al., 2017; Gray et al., 2018; Otwinowski et al., 2018; Riesselman et al., 2018; Saito et al., 2018; Wu et al., 2019; Yang et al., 2019; Shamsi et al., 2020). Limited experimental information from deep mutagenesis of one protein can be transferred through machine learning algorithms, which when combined with statistical models can better predict the effects of mutations within protein families (Gray et al., 2018; Shamsi et al., 2020). Overall, the use of statistical models offers solutions to problems with experimental mutational scans, namely limited information on epistasis and experimental noise in the data. This will continue to be a rich area for future development and improvements in the interpretation of deep mutational scans.

## Conclusion

Within the past decade, deep mutational scanning has changed how researchers approach topics of virus evolution and diversity. One is no longer limited to analyses of natural sequences, targeted mutagenesis, or isolating small numbers of clones from directed evolution in tissue culture, and instead, many thousands of mutations within viral proteins can be comprehensively assessed experimentally. This technique, whether used with live virus libraries passaged through cell culture, or expression of protein variants by yeast surface display or in mammalian cells, allows for the residue-level mapping of functional interaction sites, structural modeling, and prediction of escape mutations in response to selective pressures such as antibodies, small molecule drugs, and other therapeutics. Deep mutagenesis of viral glycoprotein spikes and host receptors also supports efforts to engineer immunogens to elicit broadly protective immunity as well as develop new treatment options, such as antibody cocktails and engineered decoy receptors. We are likely only beginning to see the tip of the iceberg for what the technology can accomplish, especially as novel selection strategies are implemented that are more quantitative. We foresee many more applications for deep mutational scanning in the future as it becomes a staple tool for exploring protein sequence landscapes.

## Author Contributions

Both authors drafted, edited the manuscript together, contributed to the article, and approved the submitted version.

## Conflict of Interest

EP was the inventor on patent filings by the University of Illinois covering soluble decoy receptors. EP was a co-founder of Orthogonal Biologics, Inc. The remaining author declares that the research was conducted in the absence of any commercial or financial relationships that could be construed as a potential conflict of interest.
